# Comparison of Ultrasound Guided and Conventional Techniques for Peripheral Venous Catheter Insertion in Pediatric Patients: A Systematic Review and Meta-Analysis of Randomized Controlled Trials

**DOI:** 10.3389/fped.2021.797705

**Published:** 2022-02-07

**Authors:** Xiulan Ye, Ming Li

**Affiliations:** Department of Pediatric, The Hospital Subordinate to Qin Hai University, Xi Ning, China

**Keywords:** children, meta-analysis, peripheral venous cannulation, ultrasonography, peripheral intravenous catheter insertion

## Abstract

**Background:**

Ultrasound guided cannulation for peripheral venous insertion is a well-established methodology. However, there has never been a systematic review completed to synthesize evidence within the pediatric population. The current systematic review and meta-analysis was completed to compare the efficacy and safety profile of ultrasound guided peripheral cannulation against the conventional palpation technique within pediatric patients.

**Methods:**

A comprehensive search was conducted within the digital databases including Medline, EMBASE, ScienceDirect, Google Scholar and Cochrane library from inception until August 2021. A meta-analysis was then completed with random-effects model and reported pooled risk ratio (RR) or standardized mean difference (SMD) with 95% confidence interval (CI).

**Results:**

In total, 9 studies were analyzed, which included 1,312 participants, and the majority of studies (5 out 9 studies) were considered high quality. Amongst efficacy outcomes, first attempt success rate had a pooled RR of 1.53 (95% CI: 1.14–2.04), overall success rate had a pooled RR of 1.13 (95% CI: 1.01–1.26), number of attempts before successful cannulation had a pooled SMD of −1.93 [95%CI: −3.44 to −0.42], time taken for successful cannulation had a pooled SMD of −0.46 [95%CI: −1.20 to 0.28], needle redirections before successful cannulation had a pooled SMD of −1.26 [95%CI: −2.47 to −0.06]. Amongst safety outcomes, venous extravasation had a pooled RR of 1.59 (95% CI: 0.99–2.54) and phlebitis had an RR of 0.31 (95% CI: 0.07–1.50).

**Conclusion:**

Within pediatric patients, ultrasound guided peripheral venous cannulation is more efficacious when compared to the conventional palpation technique.

**Systematic Review Registration:** https://www.crd.york.ac.uk/prospero/display_record.php?ID=CRD42021275305, identifier: CRD42021275305.

## Introduction

Peripheral intravenous catheter insertion is a commonly performed procedure within many healthcare settings worldwide (1). Obtaining access to the peripheral venous system is necessary for the administration of intravenous fluids and drugs, and allows for convenience when collecting blood samples (1). Traditionally, peripheral venous cannulation involves the localization of the target vessel via palpation and identification of the nearby anatomical landmarks (2). However, this technique is quite difficult for healthcare professionals to perform and is also very painful for the patients. The use of this technique for the placement of peripheral intravenous cannula within a pediatric population increases the difficulty for healthcare providers and causes much pain in the young patients. Additionally, this conventional technique for placing the peripheral venous cannula has a lower first-attempt success rate amongst the pediatric age group, ranging from 53 to 75.6% (3, 4).

Several devices have been integrated to support the insertion of the peripheral intravenous cannula. Specifically, infrared light, a constricting band, transilluminators, pressure and temperature sensors, vacuum dressing, bedside ultrasonography machines, povidone-iodine swabs, warming wraps and topical glyceryl trinitrate ointment are devices which can be used (5–7). To date, the use of ultrasonography for support of peripheral venous cannulation has been documented as the best in the literature (1).

The use of ultrasound for supporting central venous cannula insertion was first described in 1978 by Ullman and Stoelting (8). Since then, the use of ultrasound as a supportive tool for peripheral venous cannulation has become a primary focus for several randomized controlled trials (RCTs), and systematic review and meta-analyses (9, 10). This procedure has also been reviewed by the “*Agency for Healthcare Research and Quality*” in the United States of America (USA) (11). However, the use of ultrasound guided cannulation for peripheral venous insertion has not been well-established. While there has been some previous evidence, the use of ultrasonography for peripheral venous cannula insertion within pediatric patients is still relatively unexplored.

To date, only one systematic review has assessed the efficacy of ultrasonography for peripheral venous cannulation amongst children (1). However, this review included only three studies conducted within pediatric patients (1). Since then, several new trials have been published on this research question, necessitating the need for updated evidence. Hence, this review was conducted with the objective of updating the available literature and assessing the efficacy of ultrasound-guided peripheral venous catheter insertion against the conventional/traditional techniques used for peripheral catheter insertion within a pediatric population.

## Materials and Methods

### Eligibility Criteria (“PICOS”)

#### Participants

Studies completed with pediatric patients undergoing peripheral venous catheter insertion were eligible for inclusion.

#### Intervention and Comparison

Studies directly comparing the effectiveness of ultrasound guided and traditional/conventional techniques for the purpose of peripheral venous cannulation were included.

### Outcome Measures

#### Efficacy Outcomes

1. First attempt success rate
2. Overall success rate
3. Number of attempts for successful cannulation
4. Time taken for successful cannulation
5. Number of needle redirections before successful cannulation

#### Safety Outcomes

1. Venous extravasation
2. Phlebitis

### Study Design

Only randomized control trials (RCTs), individual or cluster randomized trials, were included within the systematic review. Cross over studies were excluded because of the possibility of carryover effects. Both full texts and abstracts were included within the systematic review, while unpublished literature was excluded. This study was registered at PROSPERO with number: CRD42021275305.

### Search Strategy

A comprehensive literature search was completed using the following databases: Medline, EMBASE, ScienceDirect, Google Scholar and Cochrane library. While performing the search, mixture of medical subject headings (MeSH) & free-text words were utilized. The terms used in our search strategy were as follows: “Ultrasonography,” “Peripheral Venous Catheter,” “Ultrasound guided cannulation,” “Peripheral Intravenous Catheter,” “Children,” “Paediatric,” “Randomized Controlled Trials,” “Ultrasound,” “Peripheral Intravenous Cannulation,” and “First attempt success.” The last search was completed on August 2021 and only English language studies were included. References from the retrieved studies were also searched to determine whether any studies were applicable for inclusion in the present review. The detailed search strategy is provided in the Supplementary Appendix.

### Study Selection Process

Primary screening (title, abstract & keywords screening) was completed by two independent investigators. Full texts were then retrieved for each study and selected based on the eligibility criteria. Full texts were then screened by the two investigators and those studies satisfying the inclusion criteria were included. Any disagreement during the screening procedure between the investigators was solved after discussion.

### Data Collection Process and Management

Manual data extraction was performed by the primary investigator (JC) using a pre-defined semi-structured form. Data extracted using the form were as follows: author, year of publication, country, information related to methods section such as design, setting, sample size, randomization details, participants, eligibility criteria, number of operators, static/dynamic technique, quality related information, and information related to outcome. Data was entered by the investigator and the entry was double-checked by a secondary investigator.

### Risk of Bias Assessment

The bias risk was assessed using “*Cochrane risk of Bias tool for Randomized controlled trials (RoB 2)*” (12) under the following domains:

**Domain 1:** Bias risk arising from the process of randomization
**Domain 2:** Bias risk due to deviation from the intended intervention
**Domain 3:** Bias risk arising due to missing data on outcomes
**Domain 4:** Bias risk in the outcome measurement
**Domain 5:** Bias risk in the selectively reporting outcome

Based on the rating obtained from these domains, each study was classified as having “Low bias risk,” “High bias risk,” and “Some Concerns” on the quality of evidence.

### Statistical Analysis

The meta-analysis was executed using the STATA version 14.2 (StataCorp, CollegeStation, TX, USA). Dichotomous outcomes such as success rate (overall and first attempt) and safety outcomes such as venous extravasation and phlebitis, number of outcomes and sample size in each of the groups were recorded to obtain pooled risk ratio (RR) with 95% confidence interval (CI). For efficacy outcomes, the RR is indicative of the technique with better success rate (good outcome rather than a poor outcome usually represented by RR). For safety outcomes, the RR is indicative of the technique with higher adverse events (poor outcome). Continuous outcomes such as time, number of attempts and needle redirections before successful cannulation, mean and standard deviation (SD) along with sample size in each group were recorded to obtain the standardized mean difference with 95% CI. A random effects model applying inverse variance method was used to calculate the weight of individual studies (13). Heterogeneity was statistically identified using chi square test. We also calculated the *I*^2^ statistics to quantify the level of inconsistency (13). Sensitivity analysis was done to check the robustness of pooled estimates by removing studies one by one and checking for any significant variation in the results. Publication bias and meta-regression could not be evaluated as all the outcomes were reported in <10 studies which is the minimum criteria for checking publication bias and performing meta-regression.

### Quality of Evidence

Two independent investigators assessed the risk of bias and quality of evidence for included studies using Grading of Recommendations Assessment, Development and Evaluation (GRADE) guidelines (13). The GRADE approach consists of five components: (1) Risk of bias assessment, (2) indirectness, (3) imprecision, (4) inconsistency and (5) publication bias.

**Risk of bias assessment:** “Cochrane risk of bias tool”
**Indirectness:** Assessed in terms of population, intervention, comparison, or outcomes
**Imprecision:** To determine the precision of the estimate obtained—based on sample size and confidence interval
**Inconsistency**: Evidence of heterogeneity using *I*^2^ statistic and chi square test of heterogeneity
**Publication bias**: Egger's test and funnel plot

The quality of the included studies was classified as “Very Low,” “Low,” “Moderate” and “High” based on certainty of evidence.

## Results

### Search Strategy

**Figure 1:**
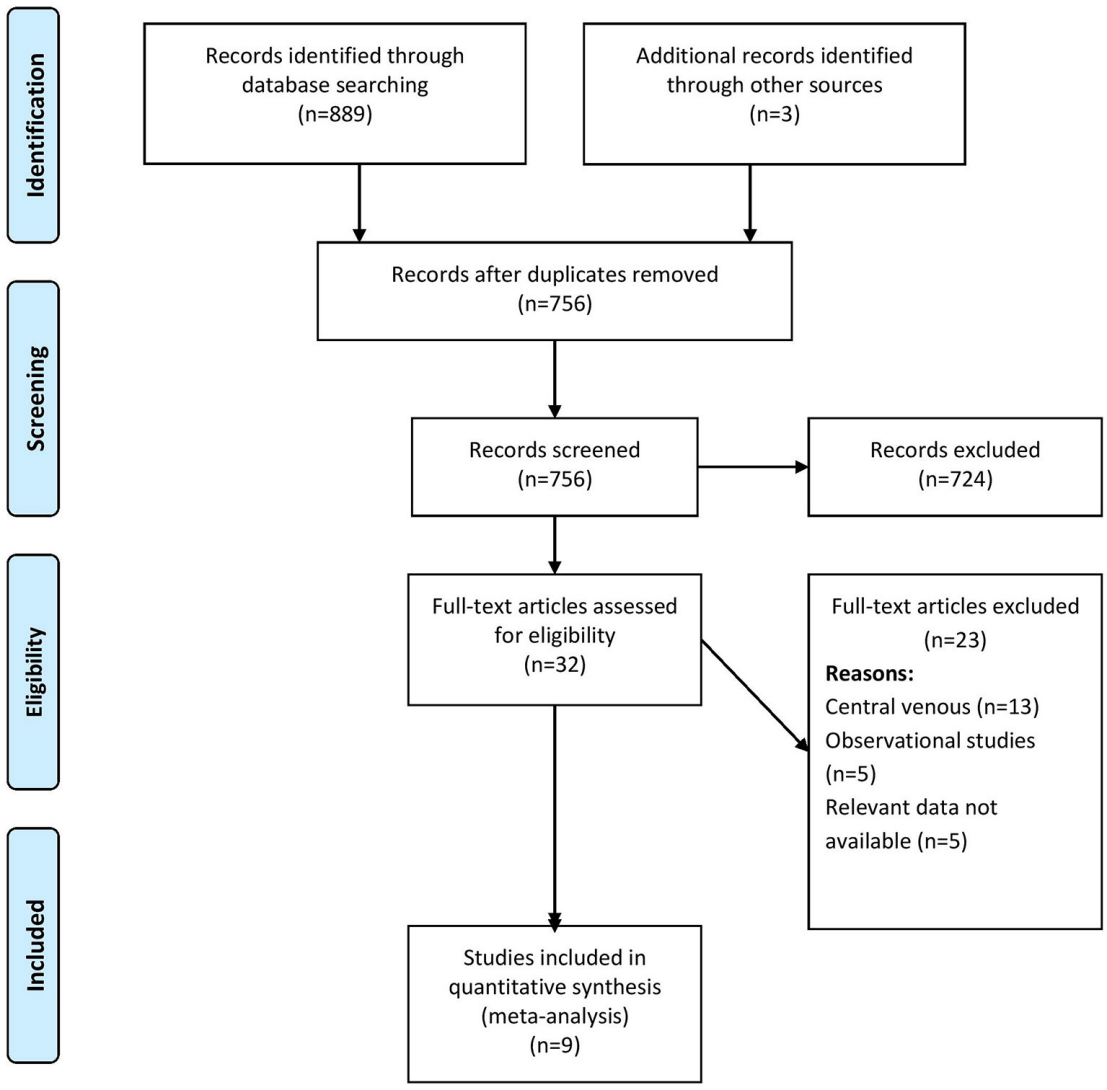
Search strategy.

The comprehensive search identified 889 records of which, when screened, 32 were found to be relevant to our review question. An additional three full texts were obtained through a manual search of the references found in the retrieved full texts. In the final stage of screening, 9 studies with 1,312 participants were included as per the eligibility criteria of the review (Figure 1) (14–22).

### Characteristics of Studies Included

Characteristics of the included studies are described in Table 1. The majority of the included studies were conducted in countries in the Americas like the USA, Canada and Brazil followed by European countries like Denmark, and France. In total, 1,312 participants were included in the analysis with sample sizes ranging from 40 to 382. Sample size within ultrasound groups ranged from 20 to 188, while sample size within conventional technique groups ranged from 20 to 194. The majority of studies (5 out of 9 studies) used the dynamic technique for ultrasound guided peripheral venous cannulation. More than half of the studies used the operating room as the study setting followed by the emergency department. Most of the studies had the anesthesiologist perform the ultrasound guided peripheral venous cannulation followed by trained nurses. The majority of the studies (5 out of 9 studies) were considered high quality as per RoB assessment checklist (Table 2).

**Table 1:** Characteristics of the included studies (*N* = 9).

**Study No**.	**References**	**Country**	**Study design**	**Sample size (ultrasound vs. conventional)**	**Study setting**	**Ultrasound technique**	**Number of operators**	**Provider**	**Outcomes assessed**	**Mean age (in years)**
1	Avelar (14)	Brazil	RCT	Ultrasound = 188 Conventional = 194	OR	Static	Two	Nurses	Overall success rate, venous extravasation, phlebitis	Ultrasound = 8.2 Conventional = 7.2
2	Bair (16)	USA	RCT	Ultrasound = 23 Conventional = 21	ED	Static	Two	Physician—USG Nurses—Catheter	First attempt success rate	Ultrasound = 1.2 Conventional = 0.7
3	Benkhadra (17)	France	RCT	Ultrasound = 20 Conventional = 20	OR	Dynamic	One	Anesthesiologist	Overall and first attempt success rate, time taken and attempts to successful cannulation	Ultrasound = 1.2 Conventional = 1.1
4	Bian (15)	China	RCT	Ultrasound = 72 Conventional = 72	OR	Static	One	Anesthesiologist	Overall and first attempt success rate, time taken, needle redirections and attempts to successful cannulation, venous extravasation	Ultrasound = 7 Conventional = 7
5	Curtis (18)	Canada	RCT	Ultrasound = 137 Conventional = 146	ED	Static & dynamic	Not reported	Nurses	First attempt success rate, time taken, needle redirections and attempts to successful cannulation	Ultrasound = 7.2 Conventional = 7.8
6	Doniger (19)	USA	RCT	Ultrasound = 25 Conventional = 25	ED	Dynamic	Two	Physician—USG Nurses—Catheter	Overall success rate, time taken, needle redirections and attempts to successful cannulation	Ultrasound = 1.8 Conventional = 2.9
7	Gopalasingam (20)	Denmark	RCT	Ultrasound = 50 Conventional = 50	OR	Dynamic	One	Anesthesiologist	Overall and first attempt success rate, time taken, needle redirections to successful cannulation	Ultrasound = NR Conventional = NR
8	Hanada (21)	USA	RCT	Ultrasound = 51 Conventional = 51	OR	Dynamic	One	Anesthesiologist	Overall and first attempt success rate, time taken to successful cannulation	Ultrasound = 12 Conventional = 8
9	Vinograd (22)	USA	RCT	Ultrasound = 83 Conventional =8 4	ED	Dynamic	One	Nurses	Overall and first attempt success rate, number of attempts to successful cannulation	Ultrasound = 5 Conventional = 5.6

*OR, Operating Room; ED, Emergency Department; USG, Ultrasonography; NR, Not reported*.

**Table 2:** Risk of bias assessment *N* = 9.

**S. No**.	**References**	**Randomization process**	**Deviation from intended intervention**	**Missing outcome data**	**Measurement of the outcome**	**Selection of the reported results**	**Overall**
1	Avelar (14)	Low	Low	Low	High	High	High
2	Bair (16)	Low	Low	Low	Low	High	Some concerns
3	Benkhadra (17)	Some concerns	Some concerns	Low	Low	Low	Some concerns
4	Bian (15)	Low	Low	Low	Low	Low	Low
5	Curtis (18)	Some concerns	Some concerns	High	Low	Low	High
6	Doniger (19)	Some concerns	Some concerns	High	Low	Low	High
7	Gopalasingam (20)	Low	Low	Some concerns	Some concerns	Low	Some concerns
8	Hanada (21)	Some concerns	Some concerns	High	High	High	High
9	Vinograd (22)	Low	Low	High	High	Low	High

### Efficacy Outcomes

#### First Attempt Success Rate

**Figure 2:**
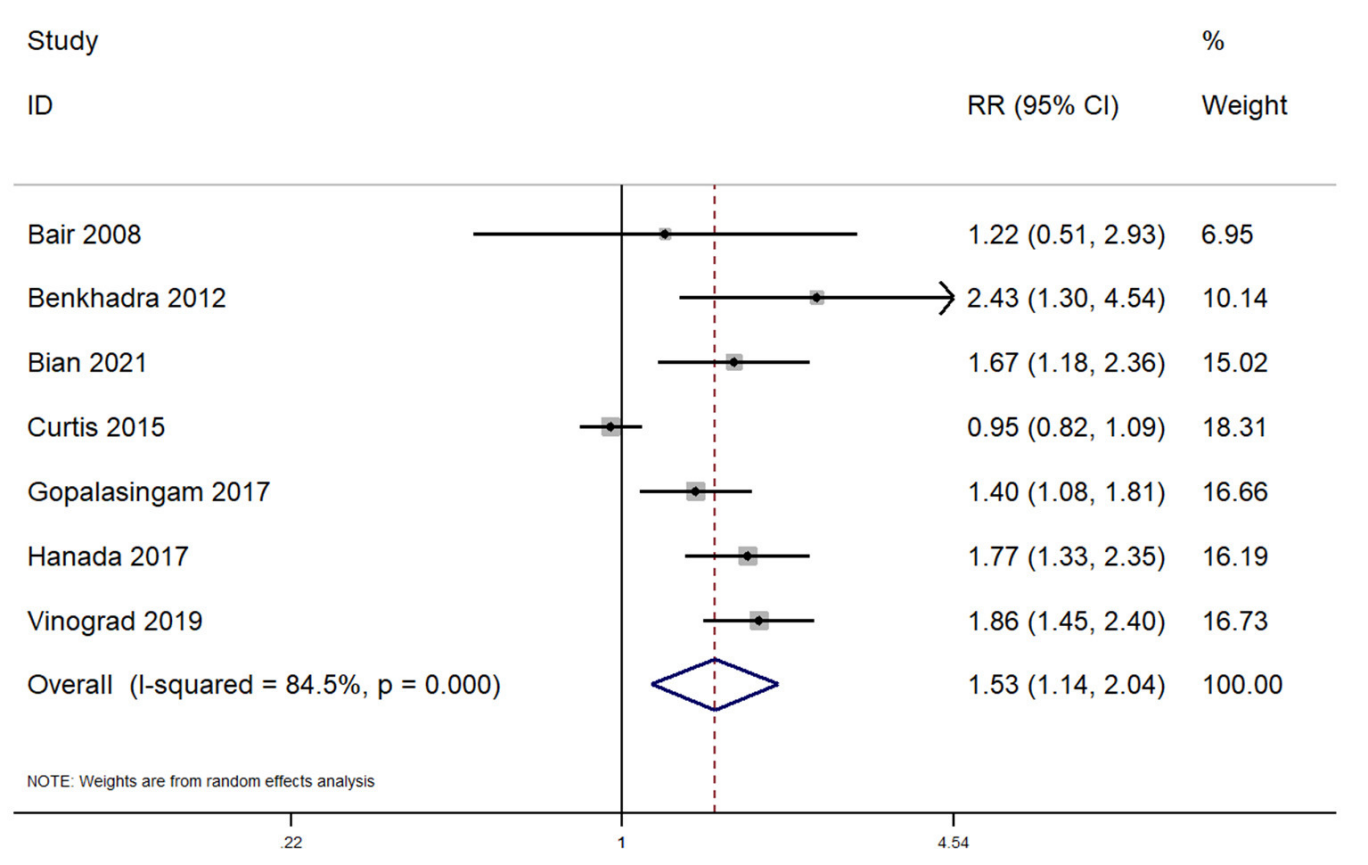
Forest plot showing the difference in first attempt success rate between ultrasound guided and conventional technique for peripheral intravenous cannulation.

In total, 7 studies reported on the first attempt success rate within pediatric patients undergoing peripheral venous cannulation. The pooled RR was 1.53 (95% CI: 1.14–2.04; *I*^2^ = 84.5%), indicating that the patients undergoing ultrasound guided peripheral venous cannulation had a significantly higher first attempt success rate when compared to patients undergoing conventional peripheral venous cannula insertion (Figure 2).

Subgroup analysis revealed that there was a significantly higher first attempt success rate when this procedure was completed across emergency departments (pooled RR = 1.63; 95%CI: 1.37–1.94). Completing the procedure in the operating room did not reveal a significantly higher first attempt success rate for ultrasound guided peripheral venous cannulation (pooled RR = 1.30; 95%CI: 0.74–2.28). Sensitivity analysis indicates the absence of single study effect on the pooled estimate of all the outcomes (Supplementary Figures 1–5). The quality of evidence was found to be low as per GRADE approach for all the outcomes.

#### Overall Success Rate

**Figure 3:**
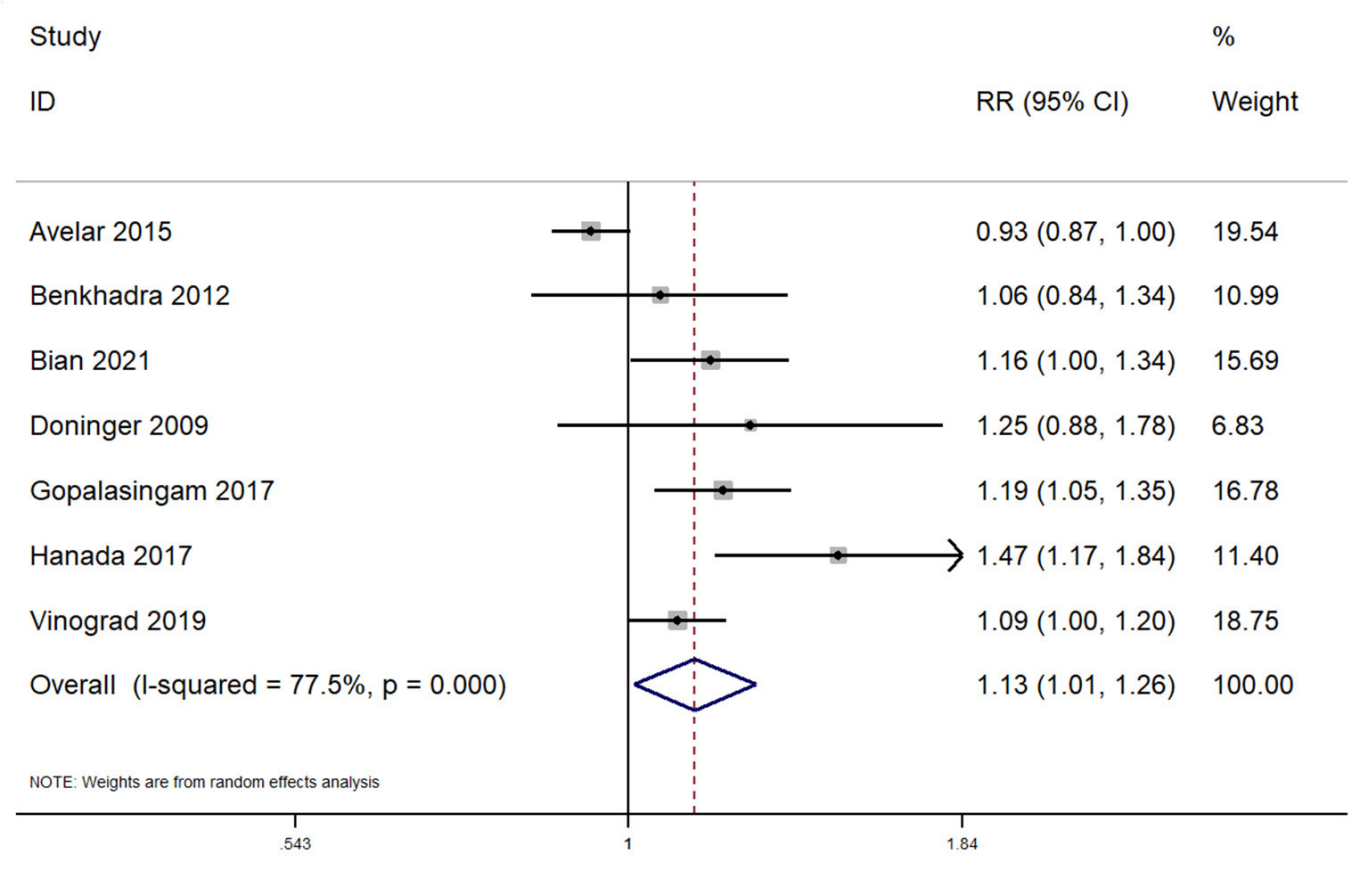
Forest plot showing the difference in overall success rate between ultrasound guided and conventional technique for peripheral intravenous cannulation.

In total, 7 studies reported on the overall success rate within pediatric patients undergoing peripheral venous cannulation. The pooled RR was 1.13 (95% CI: 1.01–1.26; *I*^2^ = 77.5%) (Figure 3). Subgroup analysis revealed that there was a significantly higher overall success rate in application of this procedure across the emergency departments (pooled RR = 1.10; 95%CI: 1.01–1.20), while completing the procedure in the operating room did not reveal a significantly higher success rate for ultrasound guided peripheral venous cannulation (pooled RR = 1.13; 95%CI: 0.97–1.33).

#### Number of Attempts for Successful Cannulation

**Figure 4:**
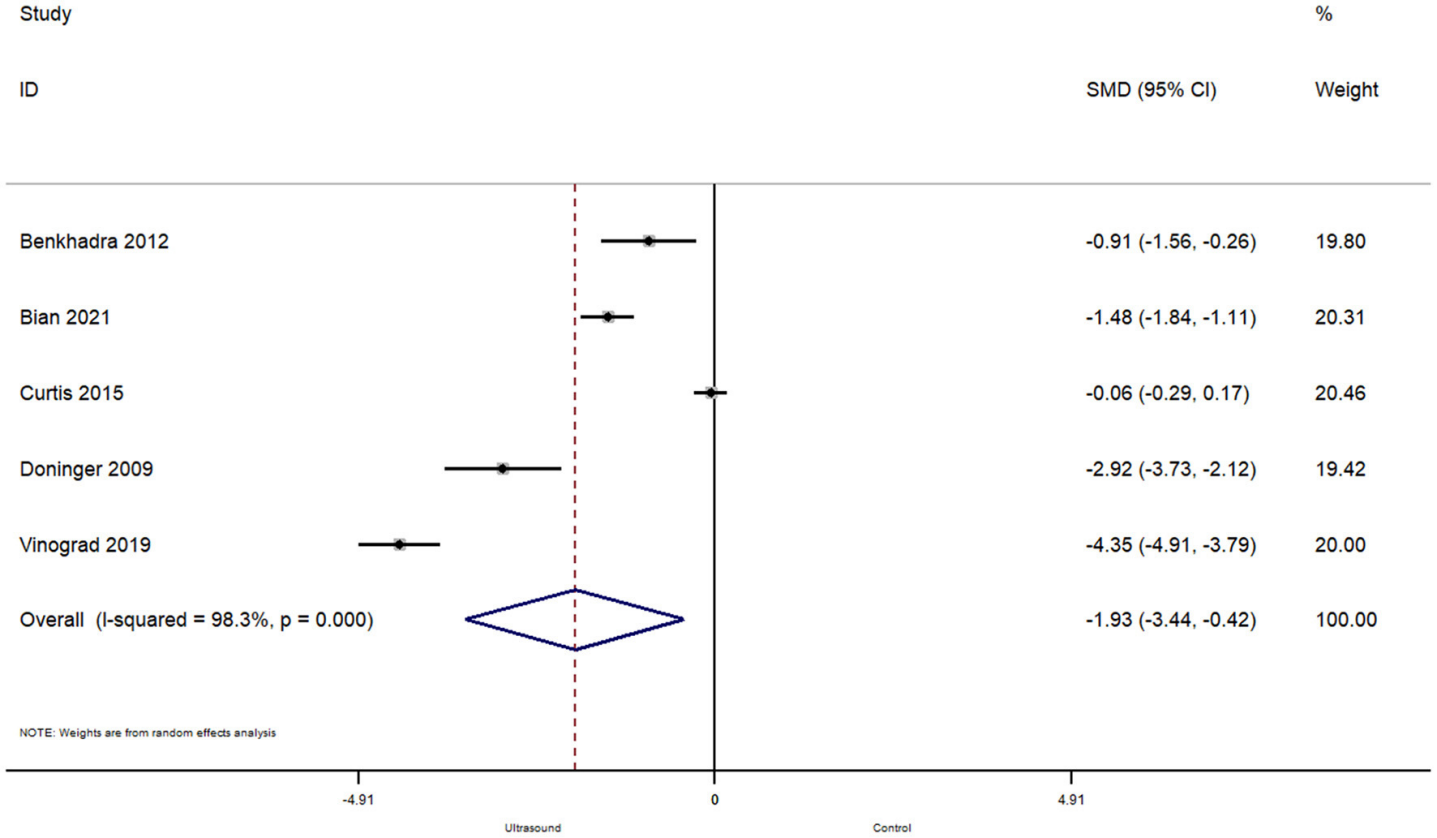
Forest plot showing the difference in number of attempts before successful cannulation between ultrasound guided and conventional technique for peripheral intravenous cannulation.

In total, 5 studies reported on the effect of ultrasound guided peripheral venous cannulation compared to conventional technique on the number of attempts for successful cannulation amongst pediatric patients. The pooled SMD was found to be −1.93 [95%CI: −3.44 to −0.42; *I*^2^ = 98.3%] and this difference was statistically significant (*p* = 0.01) (Figure 4).

Subgroup analysis revealed that when the procedure was completed in the operating room, there were significantly less attempts required for a successful cannulation (pooled SMD = −1.26; 95%CI: −1.80 to −0.72), while completing the procedure in the emergency department did not affect the number of attempts required for ultrasound guided peripheral venous cannulation (pooled SMD = −2.43; 95%CI: −5.47 to 0.61).

#### Time Taken for Successful Cannulation

**Figure 5:**
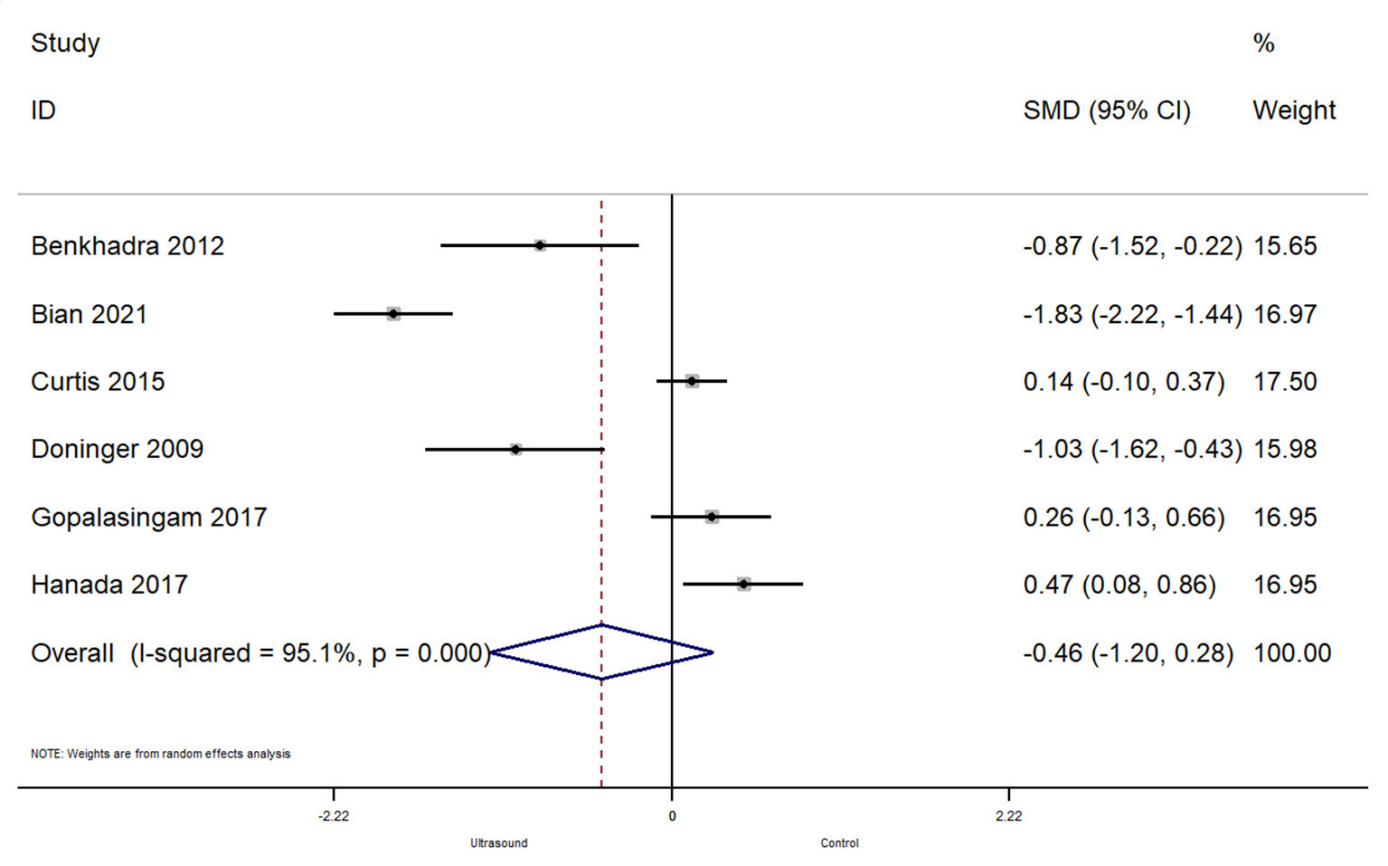
Forest plot showing the difference in time taken before successful cannulation between ultrasound guided and conventional technique for peripheral intravenous cannulation.

In total, 6 studies reported on the time taken for a successful cannulation. The pooled SMD was −0.46 [95%CI: −1.20 to 0.28; *I*^2^ = 95.1%] and this difference was not statistically significant (*p* = 0.22) (Figure 5). Subgroup analysis revealed that there were no significant differences in terms of time taken for successful cannulation within the operating room (pooled SMD = −0.49; 95%CI: −1.63 to 0.66) or emergency department (pooled SMD = −0.41; 95%CI: −1.55 to 0.72).

#### Number of Needle Redirections Before Successful Cannulation

**Figure 6:**
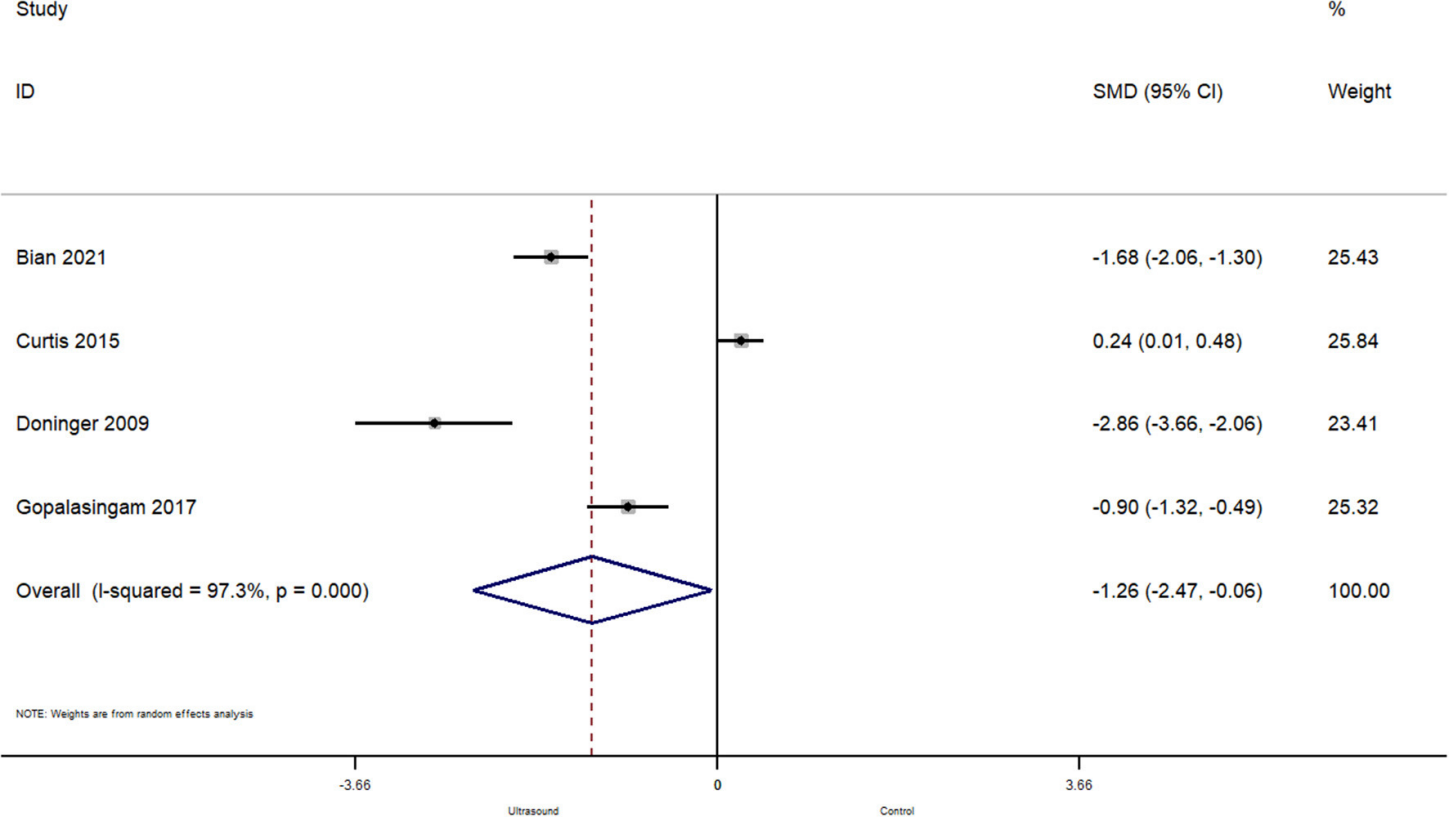
Forest plot showing the difference in number of needle redirections before successful cannulation between ultrasound guided and conventional technique for peripheral intravenous cannulation.

In total, 4 studies reported on the effect of ultrasound guided peripheral venous cannulation compared to conventional technique on the number of needle redirections before a successful cannulation within pediatric patients. The pooled SMD was −1.26 [95%CI: −2.47 to −0.06; *I*^2^ = 97.3%] and this difference was statistically significant (*p* = 0.04) (Figure 6). This indicates that the patients undergoing ultrasound guided cannulation had significantly less needle redirections before a successful cannulation compared to the conventional technique.

### Safety Outcomes

#### Venous Extravasation

**Figure 7:**
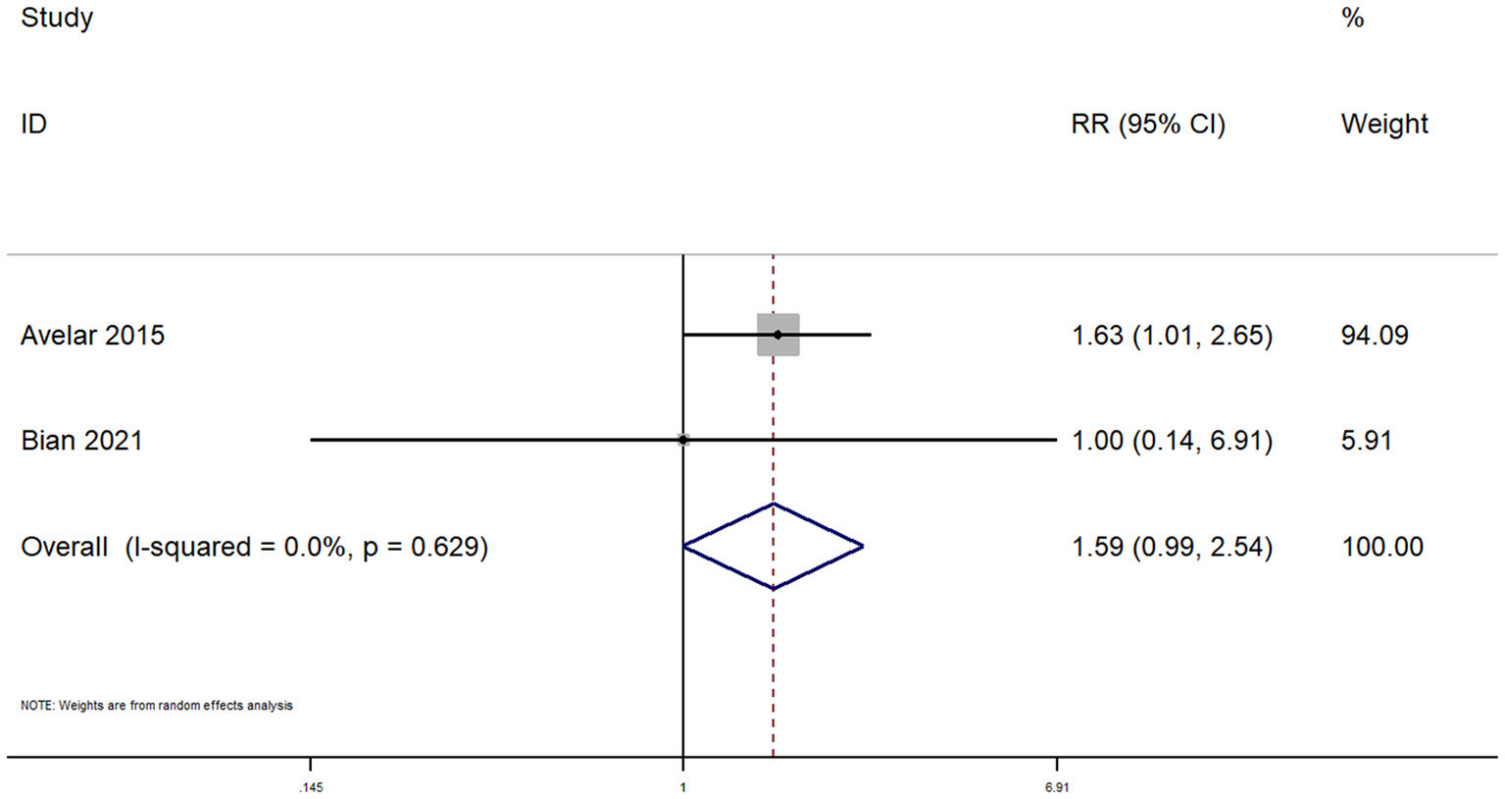
Forest plot showing the difference in venous extravasation between ultrasound guided and conventional technique for peripheral intravenous cannulation.

Only two studies reported on venous extravasation within pediatric patients undergoing peripheral venous cannulation. The pooled RR was 1.59 (95% CI: 0.99–2.54; *I*^2^ = 0%), indicating that there was no significant difference in terms of venous extravasation between the two techniques (Figure 7).

#### Phlebitis

Only one study has reported on phlebitis within pediatric patients undergoing peripheral venous cannulation in both the arms. The RR was 0.31 (95% CI: 0.07–1.50), indicating that there was no significant difference in terms of phlebitis between the two techniques.

## Discussion

Ultrasound guided cannulation has predominantly been used for central venous cannulation or arterial catheterization in its earlier years of use. Recently, this technique has become more commonly used for peripheral venous cannulation especially within difficult to access patients, such as pediatric patients. The results reported within this review reiterate the promise of ultrasound guided peripheral cannulation within the pediatric population, in which the procedure has shown a significantly higher success rate, reduction in the procedure time and number of attempts.

A previous meta-analysis examining ultrasound guided peripheral cannulation within both pediatric and adult population also found that the ultrasound guided technique was more beneficial than the conventional technique (1). However, the study reported that the ultrasound guided technique may not be useful for adults but could be considered within the pediatric population given the difficulty in visualizing their target vessels. The low number of RCTs included in that review resulted in a relatively low level of evidence. The results from this systematic review provide a more reliable indicator for the use of ultrasonography to guide the peripheral venous cannulation amongst children. Ultrasound guided peripheral venous cannulation might be a more preferable technique within the pediatric population because of the higher failure rates observed with the conventional technique in adults compared to children. These findings are because ultrasound-guided cannulation better identifies the target vessels, and collateral vasculature with real-time guidance for peripheral intravenous catheter insertion (23).

The results of this review have further confirmed the additional advantages of using ultrasound guided peripheral cannulation within pediatric patients, such as a higher success rate, fewer attempts, time taken before a successful attempt, number of needle redirections and complications (1, 9, 10). Ultrasound guided peripheral cannulation was found to be more efficacious than the conventional technique, further reiterating the need to consider this technique during routine clinical practice. However, further testing of this technique is required with large-scale and high-quality clinical trials, before it can be recommended for routine clinical practice.

In contrast to our hypothesis, ultrasound guided cannulation was found to have a greater benefit when completed within the operating room in comparison to the emergency departments or intensive care units. It is possible that the ultrasound guidance is better for patients within the operating room who benefit from obtaining intravenous access at baseline to prevent possible acute conditions like hypovolemia. Peripheral intravenous cannulation can be technically challenging especially within infants and children due to the smaller diameter of their peripheral vessels, even for experienced operators. This difficulty is increased after repeated unsuccessful attempts which may cause serious complications like hemorrhage, hematoma, venous extravasation, or phlebitis. Hence, future trials should also focus specifically on the safety component of these techniques as there was a limited number of trials included in this systematic review which reported on these outcomes within pediatric patients. The results presented here suggest that is it best to consider using ultrasound guided peripheral cannulation within pediatric patients with difficult intravenous access.

The systematic review presented here has several strengths. First, only RCTs (the highest level of evidence for reporting effectiveness of any intervention) were included within our review. In addition, most of the included studies were of high quality, thus enhancing the generalizability of our findings. While similar reviews have been published previously, this systematic review focused only on peripheral venous cannulation within pediatric patients and included almost triple the number of studies when compared to previous reviews. We have also comprehensively assessed the efficacy and safety profile of this technique by including a variety of outcomes, such as number of needle redirections and venous extravasation, which have not been reported by previous reviews. Finally, additional sensitivity analysis was performed to ensure that there was no single study effect on the overall pooled estimate.

There were a number of limitations associated with this study, one of which was that there were some major differences between the included studies. First, the number of operators (1 or 2) required to perform the ultrasound guided cannulation was not consistent between studies. Second, there was no consistency within the variation of approach used for the ultrasonographic guidance of peripheral intravenous cannulation, as some studies used a dynamic approach, while others used a static approach. Significant heterogeneity, with respect to most of the outcomes, was found between studies. This high heterogeneity could be the result of a varied study setting (ICU or ED), a difference in operators and technique. However, we could not explore these reasons given the limitation in the number of included studies which did not allow for the completion of a meta-regression. Finally, publication bias was unable to be examined due to the few efficacy and safety outcomes, which may limit the credibility of the results presented here.

Ultrasound guided peripheral cannulation, within pediatric patients, results in a higher success rate, a reduction in the overall procedure time and fewer total attempts when compared to the conventional palpation technique. Ultrasound guided peripheral venous cannulation is also more efficacious but is not different in regards to safety in comparison to the conventional technique. Future research should focus on conducting large scale RCTs within pediatric patients to further understand the benefits of this procedure and confirm the safety profile.

## Data Availability Statement

Publicly available datasets were analyzed in this study. This data can be found here: Medline, EMBASE, ScienceDirect, Google Scholar and Cochrane library from inception until August 2021.

## Author Contributions

XY conceived and designed the study and wrote the paper. XY and ML were involved in literature search and data collection, analyzed the data, and reviewed and edited the manuscript. Both authors have read and approved the final manuscript.

## Conflict of Interest

The authors declare that the research was conducted in the absence of any commercial or financial relationships that could be construed as a potential conflict of interest.

## Publisher's Note

All claims expressed in this article are solely those of the authors and do not necessarily represent those of their affiliated organizations, or those of the publisher, the editors and the reviewers. Any product that may be evaluated in this article, or claim that may be made by its manufacturer, is not guaranteed or endorsed by the publisher.

## Supplementary Material

Supplementary Figure 1 Sensitivity analysis for first attempt success rate.

Supplementary Figure 2 Sensitivity analysis for overall success rate.

Supplementary Figure 3 Sensitivity analysis for number of attempts before successful cannulation.

Supplementary Figure 4 Sensitivity analysis for time taken before successful cannulation.

Supplementary Figure 5 Sensitivity analysis for number of needle redirections.

## References

[1] Heinrichs J Fritze Z Vandermeer B Klassen T Curtis S. Ultrasonographically guided peripheral intravenous cannulation of children and adults: a systematic review and meta-analysis. Ann Emerg Med. (2013) 61:444–54.e1. 10.1016/j.annemergmed.2012.11.014 23415740

[2] Presley B Isenberg JD. Ultrasound guided intravenous access. In: StatPearls. Treasure Island, FL: StatPearls Publishing. Available online at: http://www.ncbi.nlm.nih.gov/books/NBK525988/ (accessed October 11, 2021).

[3] Lininger RA. Pediatric peripheral i.v. insertion success rates. Pediatr Nurs. (2003) 29:351–4. 14651305

[4] Black KJL Pusic MV Harmidy D McGillivray D. Pediatric intravenous insertion in the emergency department: bevel up or bevel down? Pediatr Emerg Care. (2005) 21:707–11. 10.1097/01.pec.0000186422.77140.1f 16280942

[5] Beecham GB Tackling G. Peripheral line placement. In: StatPearls. Treasure Island, FL: StatPearls Publishing. Available online at: http://www.ncbi.nlm.nih.gov/books/NBK539795/ (accessed October 11, 2021). 30969617

[6] Cantor-Peled G Ovadia-Blechman MHZ. Peripheral Vein Locating Techniques (2016) 8:83–8.

[7] Mbamalu D Banerjee A. Methods of obtaining peripheral venous access in difficult situations. Postgrad Med J. (1999) 75:459–62. 10.1136/pgmj.75.886.459 10646021 PMC1741330

[8] Ullman JI Stoelting RK. Internal jugular vein location with the ultrasound Doppler blood flow detector. Anesth Analg. (1978) 57:118. 10.1213/00000539-197801000-00024 564628

[9] Hind D Calvert N McWilliams R Davidson A Paisley S Beverley C Thomas S. Ultrasonic locating devices for central venous cannulation: meta-analysis. BMJ. (2003) 327:361. 10.1136/bmj.327.7411.361 12919984 PMC175809

[10] Mehta N Valesky WW Guy A Sinert R. Systematic review: is real-time ultrasonic-guided central line placement by ED physicians more successful than the traditional landmark approach? Emerg Med J. (2013) 30:355–9. 10.1136/emermed-2012-201230 22736720

[11] Making Health Care Safer: A Critical Analysis of Patient Safety Practices. Available online at: https://archive.ahrq.gov/clinic/ptsafety/index.html (accessed October 11, 2021).

[12] Sterne JAC Savović J Page MJ Elbers RG Blencowe NS Boutron I . RoB 2: a revised tool for assessing risk of bias in randomised trials. BMJ. (2019) 366:l4898. 10.1136/bmj.l4898 31462531

[13] Higgins, JPT, Green, S,. Cochrane Handbook for Systematic Reviews of Interventions. Available online at: https://handbook-5-1.cochrane.org/ (accessed August 22, 2021).

[14] Avelar AFM Peterlini MAS da Luz Gonçalves Pedreira M. Ultrasonography-guided peripheral intravenous access in children: a randomized controlled trial. J Infus Nurs. (2015) 38:320–7. 10.1097/NAN.0000000000000126 26339938

[15] Bian Y Huang Y Bai J Zheng J Huang Y. A randomized controlled trial of ultrasound-assisted technique versus conventional puncture method for saphenous venous cannulations in children with congenital heart disease. BMC Anesthesiol. (2021) 21:131. 10.1186/s12871-021-01349-y 33906601 PMC8077689

[16] Bair AE Rose JS Vance CW Andrada-Brown E Kuppermann N. Ultrasound-assisted peripheral venous access in young children: a randomized controlled trial and pilot feasibility study. West J Emerg Med. (2008) 9:219–24. 19561750 PMC2672282

[17] Benkhadra M Collignon M Fournel I Oeuvrard C Rollin P Perrin M . Ultrasound guidance allows faster peripheral IV cannulation in children under 3 years of age with difficult venous access: a prospective randomized study. Paediatr Anaesth. (2012) 22:449–54. 10.1111/j.1460-9592.2012.03830.x 22409596

[18] Curtis SJ Craig WR Logue E Vandermeer B Hanson A Klassen T. Ultrasound or near-infrared vascular imaging to guide peripheral intravenous catheterization in children: a pragmatic randomized controlled trial. CMAJ. (2015) 187:563–70. 10.1503/cmaj.141012 25897047 PMC4435867

[19] Doniger SJ Ishimine P Fox JC Kanegaye JT. Randomized controlled trial of ultrasound-guided peripheral intravenous catheter placement versus traditional techniques in difficult-access pediatric patients. Pediatr Emerg Care. (2009) 25:154–9. 10.1097/PEC.0b013e31819a8946 19262420

[20] Gopalasingam N Obad DS Kristensen BS Lundgaard P Veien M Gjedsted J . Ultrasound-guidance outperforms the palpation technique for peripheral venous catheterisation in anaesthetised toddlers: a randomised study. Acta Anaesthesiol Scand. (2017) 61:601–8. 10.1111/aas.12901 28485467

[21] Hanada S Van Winkle MT Subramani S Ueda K. Dynamic ultrasound-guided short-axis needle tip navigation technique vs. landmark technique for difficult saphenous vein access in children: a randomised study. Anaesthesia. (2017) 72:1508–15. 10.1111/anae.14082 28983903

[22] Vinograd AM Chen AE Woodford AL Fesnak S Gaines S Elci OU . Ultrasonographic guidance to improve first-attempt success in children with predicted difficult intravenous access in the emergency department: a randomized controlled trial. Ann Emerg Med. (2019) 74:19–27. 10.1016/j.annemergmed.2019.02.019 31126618

[23] Hansen MA Juhl-Olsen P Thorn S Frederiksen CA Sloth E. Ultrasonography-guided radial artery catheterization is superior compared with the traditional palpation technique: a prospective, randomized, blinded, crossover study. Acta Anaesthesiol Scand. (2014) 58:446–52. 10.1111/aas.12299 24588456

