# Supplementary figures and images for: Comparison of Ultrasound Guided and Conventional Techniques for Peripheral Venous Catheter Insertion in Pediatric Patients: A Systematic Review and Meta-Analysis of Randomized Controlled Trials
Source: Front Pediatr. 2022 Feb 7;9:797705. doi: 10.3389/fped.2021.797705 (PMC8859100; doi:10.3389/fped.2021.797705)

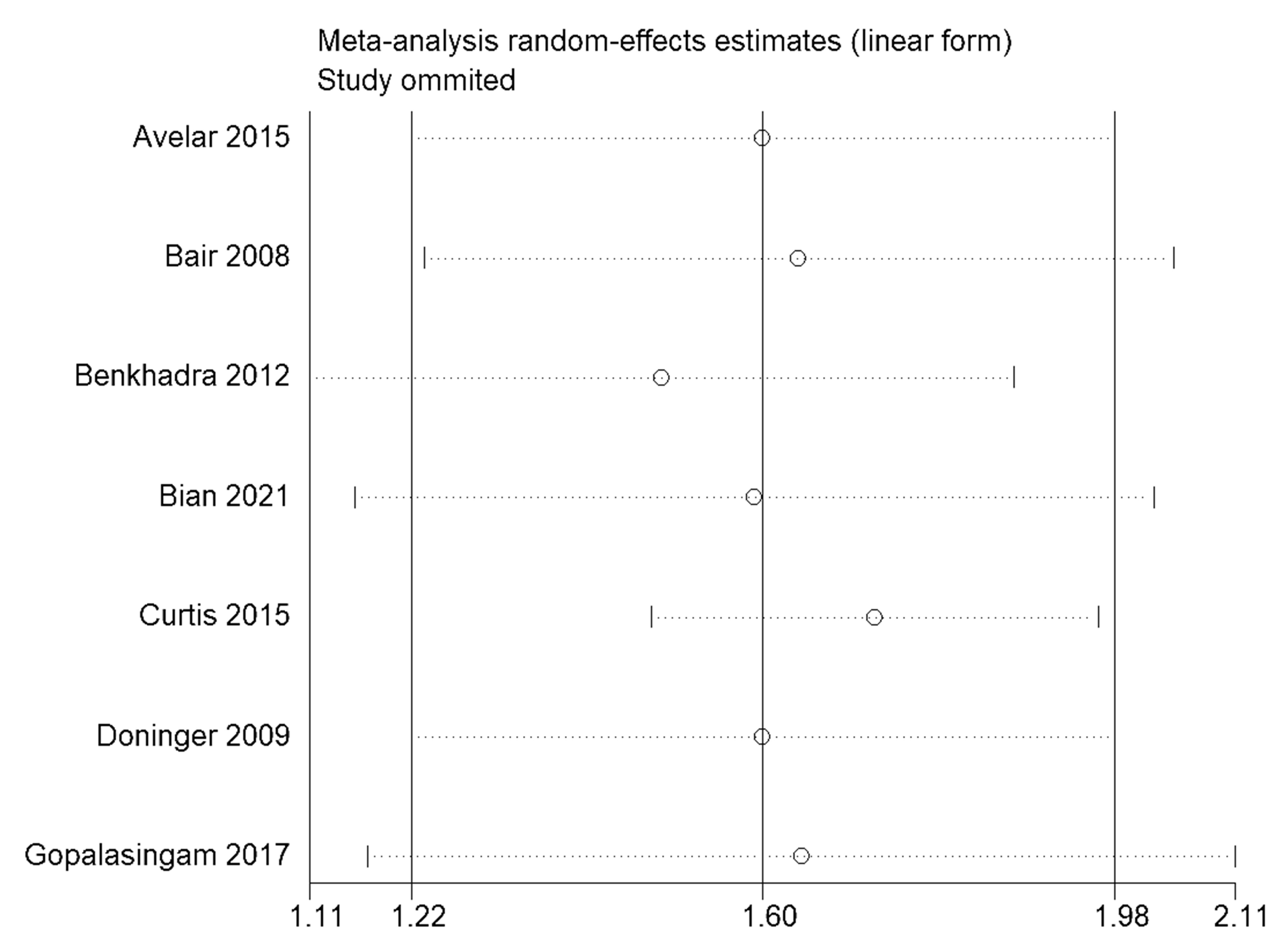

Supplement: Supplementary Figure 1 — Sensitivity analysis for first attempt success rate. [file Image_1.TIF]

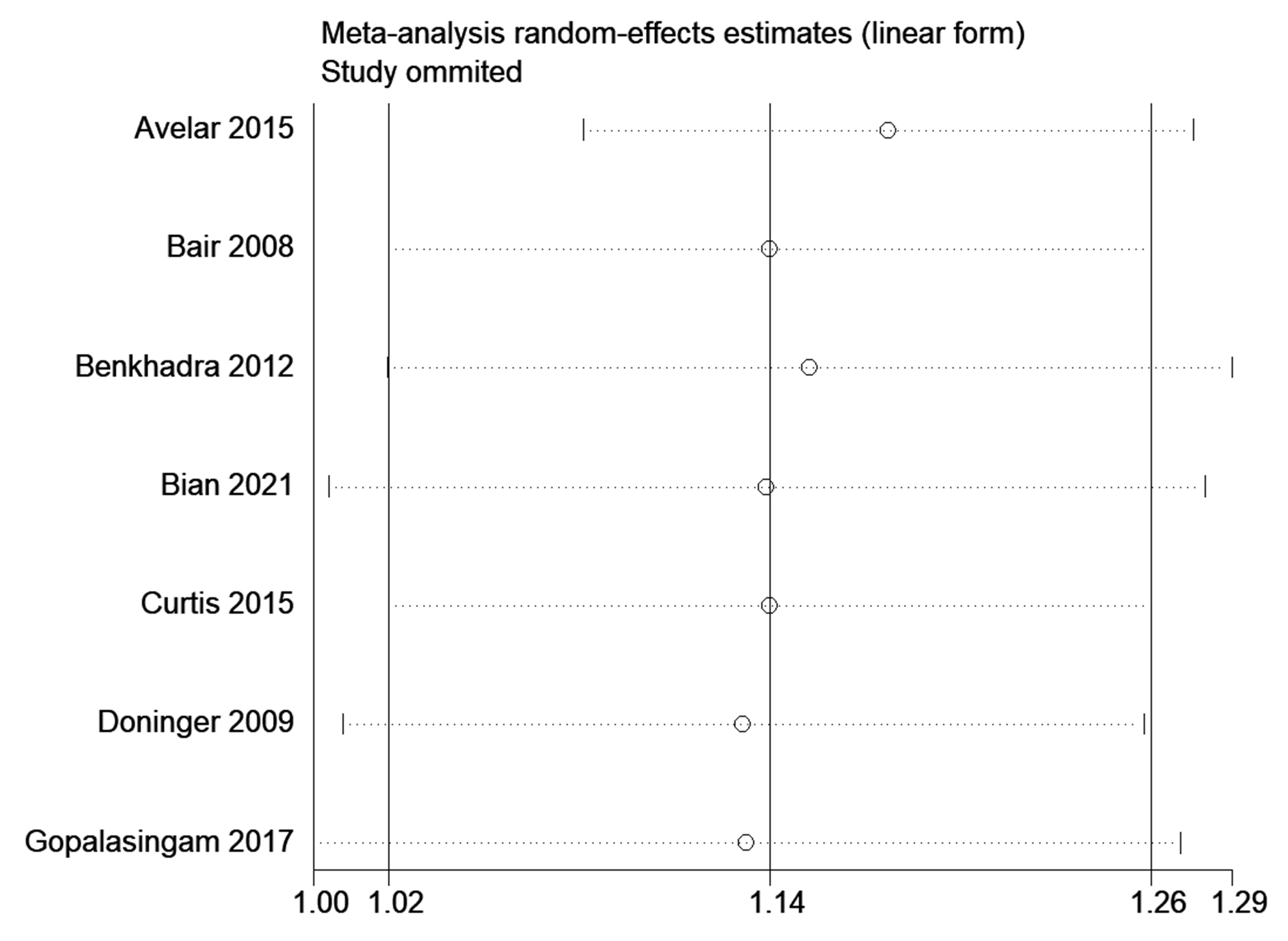

Supplement: Supplementary Figure 2 — Sensitivity analysis for overall success rate. [file Image_2.TIF]

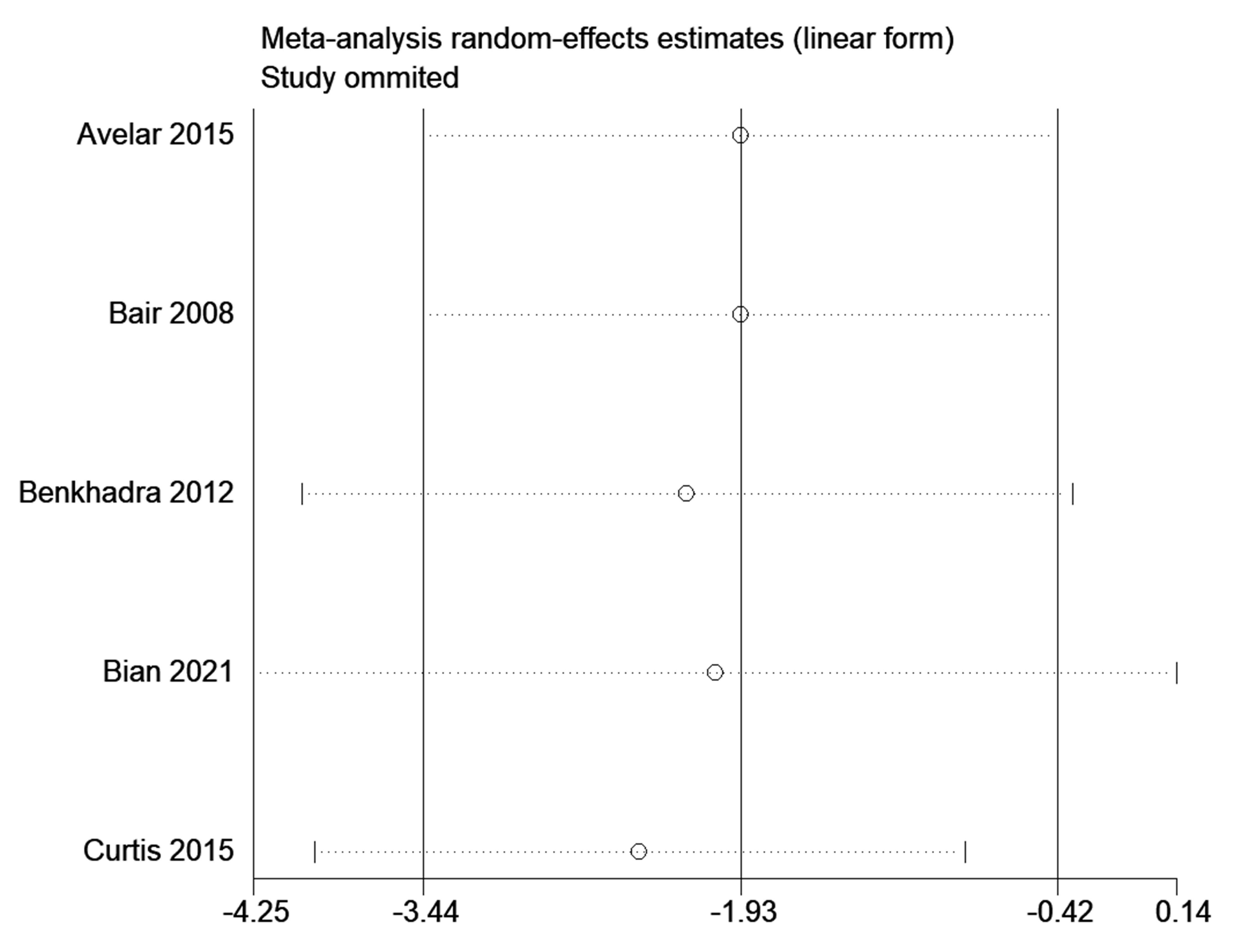

Supplement: Supplementary Figure 3 — Sensitivity analysis for number of attempts before successful cannulation. [file Image_3.TIF]

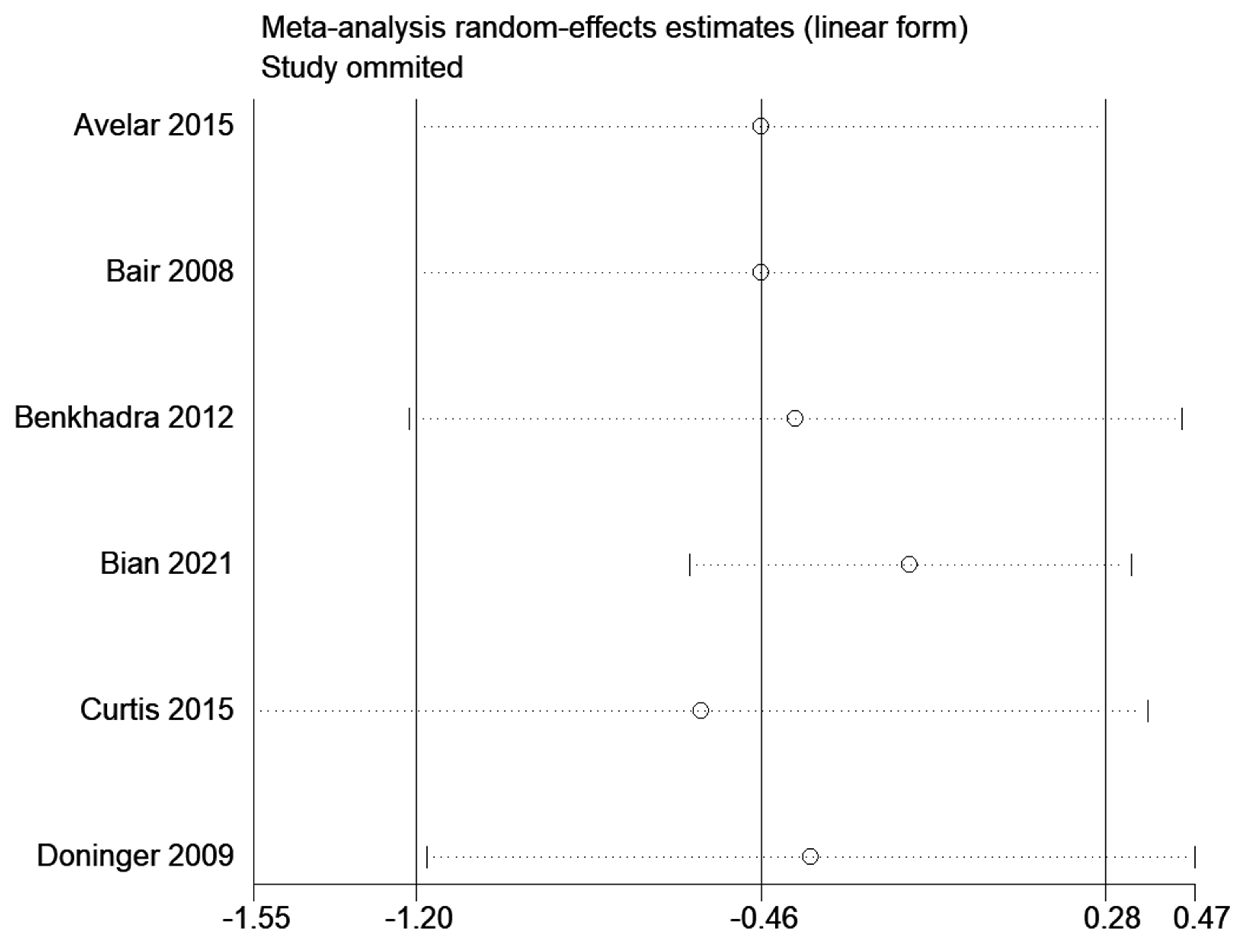

Supplement: Supplementary Figure 4 — Sensitivity analysis for time taken before successful cannulation. [file Image_4.TIF]

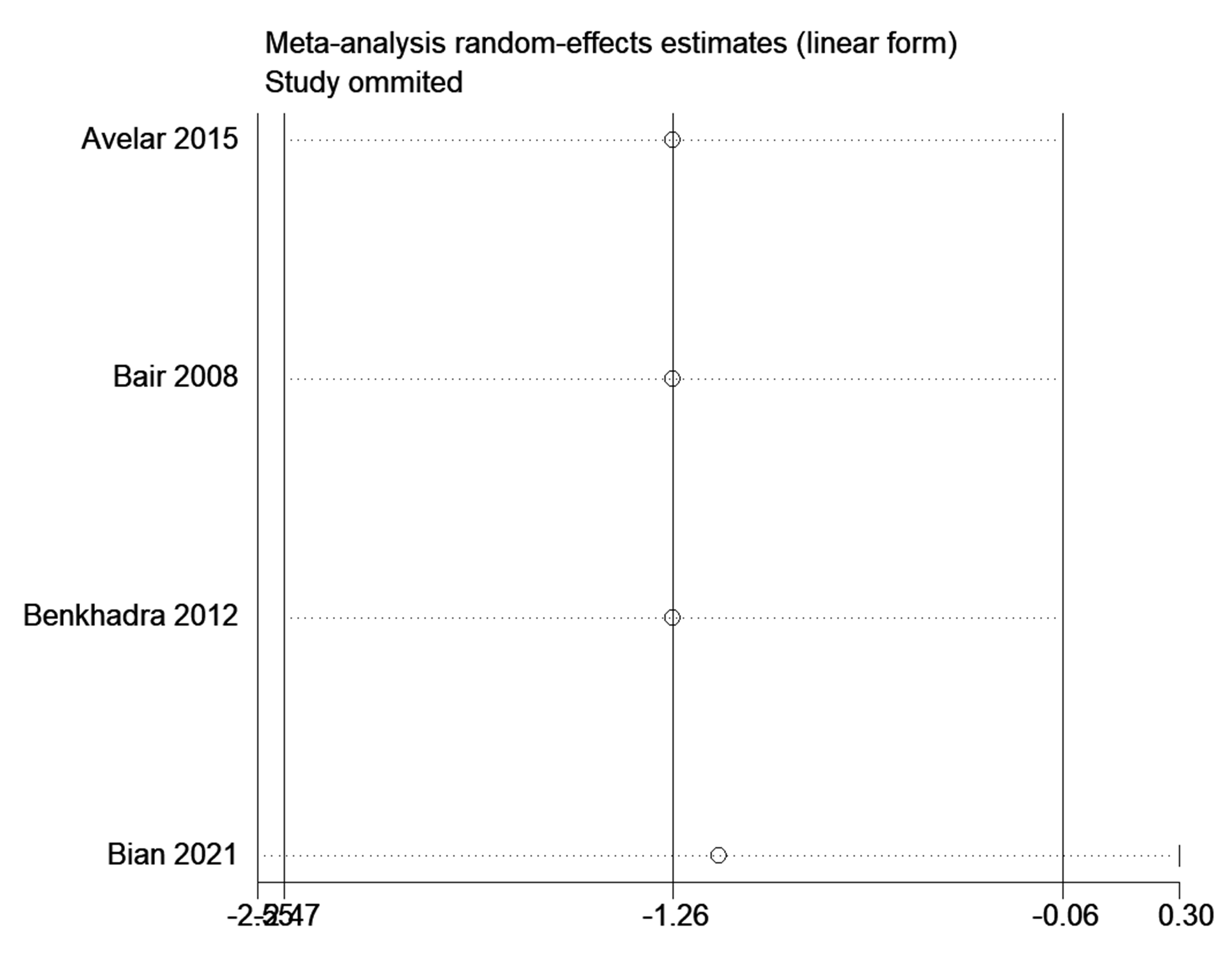

Supplement: Supplementary Figure 5 — Sensitivity analysis for number of needle redirections. [file Image_5.TIF]
