# Supplementary material for: Comparison of Ultrasound Guided and Conventional Techniques for Peripheral Venous Catheter Insertion in Pediatric Patients: A Systematic Review and Meta-Analysis of Randomized Controlled Trials
Source: Front Pediatr. 2022 Feb 7;9:797705. doi: 10.3389/fped.2021.797705 (PMC8859100; doi:10.3389/fped.2021.797705)
Supplement: Supplementary file 6 [file Table_1.DOCX]

**Search strategy:**

**EMBASE:**

('child'/exp OR 'child' OR 'children' OR 'adolescent'/exp OR 'adolescent' OR 'teenager') AND ('ultrasound'/exp OR 'phonophoresis' OR 'radiation, ultrasonic' OR 'sonication' OR 'sonification' OR 'ultra sound' OR 'ultrashell' OR 'ultrasonic' OR 'ultrasonic energy' OR 'ultrasonic irradiation' OR 'ultrasonic measurement' OR 'ultrasonic sound' OR 'ultrasonic wave' OR 'ultrasonic waves' OR 'ultrasonics' OR 'ultrasound' OR 'ultrasound radiation') AND ('peripheral venous catheter'/exp OR 'abbocath' OR 'angiocath' OR 'angiocath autoguard' OR 'bd angiocath' OR 'bd angiocath autoguard' OR 'bd angiocath-n autoguard' OR 'bd cathena safety iv catheter' OR 'bd insyte' OR 'bd insyte autoguard' OR 'bd insyte-n' OR 'bd insyte-n autoguard' OR 'bd insyte-w' OR 'bd nexiva' OR 'bd nexiva bd q-syte' OR 'bd nexiva diffusics' OR 'clarivein' OR 'insyte autoguard' OR 'intracath (peripheral venous catheter)' OR 'introcan' OR 'jelco' OR 'midline catheter kit' OR 'neoflon' OR 'powerstick' OR 'supercath 5' OR 'surflash' OR 'volumeview' OR 'peripheral intravenous catheter' OR 'peripheral intravenous catheterization kit' OR 'peripheral vascular catheter' OR 'peripheral venous access catheter' OR 'peripheral venous catheter' OR 'peripheral venous line') – **99 results**
